# Osteoporosis in Light of a New Mechanism Theory of Delayed Onset Muscle Soreness and Non-Contact Anterior Cruciate Ligament Injury

**DOI:** 10.3390/ijms23169046

**Published:** 2022-08-12

**Authors:** Balázs Sonkodi, Rita Bardoni, Gyula Poór

**Affiliations:** 1 Department of Health Sciences and Sport Medicine, Hungarian University of Sports Science, 1123 Budapest, Hungary; 2 Department of Biomedical, Metabolic and Neural Sciences, University of Modena and Reggio Emilia, 41121 Modena, Italy; 3 National Institute of Locomotor Diseases and Disabilities, 1023 Budapest, Hungary; 4 Section of Rheumatology and Physiotherapy, Department of Internal Medicine and Haematology, Semmelweis University, 1085 Budapest, Hungary

**Keywords:** osteoporosis, delayed onset muscle soreness, non-contact injury, Piezo2 ion channel, channelopathy, quad-phasic non-contact injury model

## Abstract

Osteoporosis is a disorder, with a largely unknown pathomechanism, that is often marked as a “silent thief”, because it usually only becomes undisguised when fractures occur. This implies that the pathological damage occurs earlier than the sensation of pain. The current authors put forward a non-contact injury model in which the chronic overloading of an earlier autologously microinjured Piezo2 ion channel of the spinal proprioceptor terminals could lead the way to re-injury and earlier aging in a dose-limiting and threshold-driven way. As a result, the aging process could eventually lead the way to the metabolic imbalance of primary osteoporosis in a quad-phasic non-contact injury pathway. Furthermore, it is emphasised that delayed onset muscle soreness, non-contact anterior cruciate injury and osteoporosis could have the same initiating proprioceptive non-contact Piezo2 channelopathy, at different locations, however, with different environmental risk factors and a different genetic predisposition, therefore producing different outcomes longitudinally. The current injury model does not intend to challenge any running pathogenic theories or findings, but rather to highlight a principal injury mechanism.

## 1. Introduction

Osteoporosis is a disorder associated with fragility due to the systemic microdeterioration of the bone tissue and is often marked as a “silent thief”, because it usually only becomes undisguised when fractures occur [1,2]. Delayed onset muscle soreness (DOMS) is defined by delayed onset soreness, muscle stiffness, loss of force-generating capacity, reduced joint range of motion and decreased proprioceptive function [3]. Non-contact anterior cruciate ligament (NC-ACL) injuries comprise approximately 3/4 of all anterior cruciate ligament (ACL) injuries that occur when the ligament fibres are stretched or partially or completely torn on a non-contact (NC) basis [4]. None of the above disorders have an entirely known pathomechanism. However, the pathological damage is suspected earlier than the sensation of pain in all three cases, which implies differing lengths of silent pathological periods prior to pain manifestation. New hypotheses might provide us with an explanation for these earlier “silent” courses, not to mention their possible common origin. The objective of this current study is to apply the quad-phasic non-contact injury model (Table 1) [5] to osteoporosis where the primary microdamage is suggested to be the same proprioceptive terminal Piezo2 channelopathy, like in other NC injuries such as DOMS or NC-ACL. It is important to note that the current opinion of the authors does not intend to challenge any earlier pathogenic theories or findings, but rather highlights a principal non-contact injury mechanism that could eventually evolve into osteoporosis, if re-injury, environmental risk factors or genetic predisposition are present. Furthermore, this manuscript intends to facilitate an interdisciplinary approach between physiology, pathology, neuroscience, endocrinology, rheumatology, orthopaedics and immunology in order to better understand and improve the management of osteoporosis, as well as to find even more tailored therapeutic interventions.

## 2. Primary Non-Contact Injury Phase

The acute compression axonopathy theory of DOMS puts forward that it is a dichotomous muscular NC injury where the primary microdamage evolves in the Type Ia proprioceptive terminal of the muscle spindle (MS) [6]. Unaccustomed or strenuous eccentric contractions lead the neuromodulator muscle and neighbouring tissue cells to hyperexcitation by the Cox2-PGE2 and Cox2-bradykinin-nerve growth factor (NGF) pathways [6]. However, when the performance of muscle cells is not sustained sufficiently, then a cognitive-demand-induced acute stress response (ASR) could kick in as a driver [6,7]. Nevertheless, the terminal arbours of the Type Ia proprioceptive sensory nerves in the MS could undergo a mechano-energetic microinjury during an ASR [6]. The eccentric-contractions-derived superposition of compression forces could have relevance in this microdamage in a dose-limiting, threshold-driven manner [6]. The notion of the existence of this distinct somatosensory terminal impairment mechanism could be learnt from chemotherapy, and it could evolve in an acute and chronic way without causing classical Wallerian degeneration [6,8]. Furthermore, it is proposed that the critical gateway to pathology could be a silent and transient channelopathy of Piezo2 ion channels [5,9]. It is noteworthy that these Piezo2 ion channel microinjuries could remain pain-free in cases when the superseding lengthening contractions are abruptly terminated right after the initiation of channelopathy [9].

It is important to note that bones and muscles construct a continuum when it comes to the neuromodulation and hyperexcitation of the proprioceptive system, not to mention their similar signalling pathways [10]. Furthermore, the proposed ASR is also bone-derived, namely osteocalcin-induced [6,11]. Correspondingly, another new theory for NC-ACL injury argues that the primary injury is also a silent proprioceptive terminal lesion, but in the periosteum of the medial proximal tibia, like in the case of DOMS [6]. Indeed, similar primary afferent mechano-sensitive encapsulated endings could be found in the spine as well [12,13]. Only the secondary harsher tissue damage, derived from the impaired proprioceptive capacity, could entail actual ACL injury [10]. It is noteworthy that this new hypothesis could also explain the significant sex differences in the epidemiology of NC-ACL as neuronal, namely by the pre-ovulatory transient luteinising-hormone-derived NGF-tropomyosin receptor kinase A (TrkA) signalling pathway [5,10]. This signalling axis could further facilitate the noxious hyperexcitation of the sensory terminals, rendering it even more prone to microinjury under an ASR [10]. Moreover, Piezo2 ion channels are mainly located on proprioceptive and tactile fibres, but a significant proportion of Aδ mechano-nociceptors in the bone tissue express these channels as well [14]. Accordingly, recent animal research shows that a group of bone afferent sensory neurons that express Piezo2 and co-express TrkA are the ones that have high affinity for NGF [14]. Moreover, Piezo2 has a role in bone afferent neurons when it comes to noxious mechanical stimulation, not to mention its role in NGF-induced bone afferent sensitisation to mechanical stimulation [14], substantiating the hypothesis that Piezo2 is a critical player in these pathologies [5,6,9,10].

The proprioceptive signalling of MSs and Golgi tendon organs (GTOs) is essential for the non-autonomous morphologic restoration of microfractured bones or remodelling [15,16]. Moreover, it is suggested that the primary transient proprioceptive terminal Piezo2 microinjury concomitant with microfractures impairs the static phase firing encoding of the stretch reflex [7,10,17,18] and correspondingly, this impairment could be realigned by the dynamic encoding of MSs and GTOs [19,20]. Hence, proprioceptive sensory neurons are proposed to have a role not only in the guidance of growth and regeneration, but also remodelling [6,9,10].

## 3. Secondary Injury Phase

The secondary phase of NC injuries is a harsher tissue damage in a subluxated position due to impaired proprioception [6,10,21]. C sensory fibre contribution comes into play in this phase, providing the temporal summation of pain [22]. The parallel that vertebral compression fractures [10], NC-ACL injury [10], DOMS [6,9] and osteoporosis may pertain to the same primary proprioceptive microinjury in a dichotomous NC injury mechanism could carry relevance, especially if we consider the theory that the termination of lengthening contractions and the lack of harsher secondary tissue damage or compression fractures after the transient primary injury keep these microinjuries and concomitant microcracks pain-free [9,10].

Notably, it is hypothesised that the repetitive recurrence of the primary microdamage could initiate the tertiary injury phase even in the absence of the secondary injury phase [9]. However, C-fibre involvement with harsher tissue damage in the form of fractures, which is the equivalent of the secondary injury phase, must come into play in a later stage, when pain sensation becomes part of the clinical picture.

## 4. Tertiary Injury Phase

The new DOMS and NC-ACL theories also suggest a tertiary phase of the initial primary Piezo2 channelopathy [9,10]. In the case of DOMS, it is called the repeated bout effect (RBE), while in the case of NC-ACL it is re-injury and osteoarthritis (OA) [6,9,10,23]. It is noteworthy that earlier aging of the knee joint is the consequence of NC-ACL injury and that 4/5 of cases develop into OA [24]. In particular, recent findings of OA research are interesting in regard to osteoporosis. Acid-sensing ion channel 3 (ASIC3) plays a crucial role in the secondary hyperalgesia of joint inflammation in OA rats [25,26], as it does in osteoporosis [1], but not in primary hyperalgesia [25]. Correspondingly, the gradual upregulation of ASIC3 channels detected in dorsal root ganglion (DRG) primary afferent neurons of knee joints in OA and the activated immune cells in neural tissues are key players in the development of secondary hyperalgesia and the degeneration process of OA [25]. The current authors propose that the repeated or chronic Piezo2 microdamage-induced upregulation of ASIC3 is the equivalent of the tertiary injury phase [23,25,27]. The activation and upregulation of ASIC3, together with transient receptor potential cation channel subfamily V member 1 (TRPV1), are also driven by the release of inflammatory mediators, NGF and the osteoclast hyperactivity, causing a decrease in extracellular pH [28].

Accordingly, Lin et al., demonstrated in mice that ASIC3 also contributes to mechano-transduction in proprioceptors [29], as Piezo2 primarily does [30]. Indeed, ASIC-like acid-induced inward currents persisted in proprioceptive ASIC3 DRG neurons under this pathological environment [29]. Moreover, ASIC3 channels could also have a longitudinal role in memory formation [31], once activated N-methyl-D-aspartate (NMDA) receptors open memory pathways, including immune memory on the spinal dorsal horn as a consequence of the primary Piezo2 microdamage [7,9,10]. In summary, these persistent ASIC-like currents are suggested to be evoked in osteoporosis as well and sustained by the subthreshold-imbalanced Piezo Ca^2+^ currents due to the Piezo2 microinjury-derived “leakiness” [5,9].

Longitudinally, the microinjured Piezo2 channels in the periosteum, or spine, could facilitate the gradual upregulation of ASIC3 channels in the DRG under a chronic overloaded environment [23]. Furthermore, another consequence of this Piezo2 microdamage on the periphery of OA could be the upregulation of Piezo1 in the affected neuromodulator tissues, such as the chondrocytes in a feed-forward mechanism [32]. It is noteworthy that Piezo1 channels contribute to detecting cell alignment based on their shear stress sensor capability [33,34]; therefore, this signalling could also be essential for remodelling [23]. Furthermore, it should not be excluded that Piezo1 microinjury could evolve into Piezo2 microinjury gradually in osteoporosis, as is suggested in the paradox continuum of initially pain-free dry eye disease into neuropathic corneal pain [5], not to mention that the “leakiness” to subthreshold-imbalanced Piezo Ca^2+^ currents due to these Piezo microinjuries could explain the “calcium stealing” from bones for years before pain evolves due to fractures [1].

This degenerative process of the tertiary phase is costly in terms of neuro-energetics, because it uses progressively more synaptic connections and secondary compensatory microcircuits in the central nervous system (CNS), and as a result facilitates augmented neuroinflammation in the CNS and the upregulation of cytokines and inflammatory mediators on the periphery [6,7,10], not to mention that bone sensory innervation increases with age [1,35]. Consequently, the progressive activation of NMDA receptors, the activation of microglia, the overexpression of ASIC3 and TRPV1 ion channels on nociceptive sensory neurons and the increase in TrkA^+^ nerve fibres are the consequence of this third NC injury phase in osteoporosis, leading to changes in spinal cord dorsal horn circuits, as is explained in the narrative review of Mattia et al. [1]. Central sensitisation mechanisms further involve the potentiation of NMDA receptor function, the activation of both microglia and astrocytes and the release of peptides, such as substance P [1,28]. Synaptic gamma-aminobutyric acid (GABA)-mediated inhibition (both at the pre- and post-synaptic level [36]) could also be impaired longitudinally by changes in GABA vesicular transport, GABA re-uptake, the alteration of intracellular chloride concentration and the modification of GABA receptor composition [37,38,39]. Indeed, aging women with osteoporotic fracture have significantly lower GABA levels [40]. However, in the absence of secondary harsher tissue injury or compression fractures and C-fibre temporal summation, all of these microfractures and concomitant Piezo channelopathies could remain silent [9].

## 5. Quadric Injury Phase

It is important to note that the aging process, also termed inflammaging, could further augment the tertiary degeneration process both in the CNS and on the periphery, and it is described by the current authors as the quadric phase of the primary proprioceptive Piezo2 microinjury [7,10], not to mention that the aging-augmented processes could lead to suppressed sensory signalling, e.g., the NGF-TrkA axis [41] and osteocalcin [42], paving the way to osteoporosis. The chronic microinjury of spinal proprioceptive Piezo2 and the aging process together could accelerate the imbalance of the aforementioned alignment process and could lead to osteoporotic fractures, in parallel with the imbalance of osteoblastic and osteoclastic activity [43]. It is important to remark that proprioceptive sensory function is progressively diminished by aging [44].

## 6. Future Targeted Therapeutic Interventions

It is important to note that the remodelling process involves the role of interleukin-6 in DOMS, anterior cruciate ligament injury, OA and osteoporosis as well [45,46,47,48]. Unfortunately, an increased rate of bone remodelling will result in lower bone mass [47]. Accordingly, the elevated interleukin-6 in the above chronic conditions is suggested to increase the bone remodelling rate. Indeed, the reduction in the elevated serum levels of interleukin-6 in the above chronic conditions by monoclonal antibodies against the interleukin-6 receptor or by oxygen-ozone therapy seems to be a promising therapeutic strategy for the future [46,47].

It was proposed that the chronic somatosensory Piezo2 channelopathy or the permanent unwanted leakiness of these ion channels could be interpreted as “part of wound healing is kept alive” permanently instead of transiently [5]. Furthermore, it was hypothesised that if canonical Wnt signalling is inhibited by interleukin-6, then the osteogenic differentiation will be blocked [47]. On the contrary, if the non-canonical Wnt signalling pathway is induced by interleukin-6, then osteogenic differentiation will be promoted [47]. Correspondingly, mesenchymal stem cell therapy in combination with hyperbaric oxygenation treatment is a promising therapeutic approach [49] in order to promote adequate “wound healing” in chronic Piezo2 channelopathy.

It is noteworthy that not only is pain missing in loss-of-function mutations in Piezo2, but sensitisation as well [50]. However, the paradox exists in osteoporosis, like in dry eye disease [5], that it is a pain-free condition in most cases, although it could be regarded as a pain condition as well [51]. As noted earlier, C-fibre contribution, that is, the secondary injury phase, and noxious mechanical stimulation are needed for chronic nociceptive and neuropathic pain evolvement, but the primary injury phase is silent, as is the tertiary injury phase in the absence of the secondary injury phase. Therefore, not only is the acute or chronic trauma-related pain management important [51,52], but the prevention of re-injury and the prevention of overloading Piezo2 channelopathy is recommended [23].

## 7. Limitations

The current opinion manuscript does not represent a systemic review with limited evidence, but rather provides a theory about a possible parallel between the quad-phasic non-contact injury model and the pathomechanism of osteoporosis. Furthermore, it intends to facilitate an interdisciplinary approach between different disciplines (including physiology, pathology, neuroscience, endocrinology, rheumatology, orthopaedics and immunology), in order to better understand the pathomechanism and improve the management of osteoporosis.

## 8. Conclusions

In summary, the current authors put forward that the chronic overloading of the previously microinjured Piezo2 of the spinal proprioceptor terminals could lead the way to re-injury and earlier aging in a dose-limiting and threshold-driven way [10], but the aging process could eventually lead the way to the metabolic imbalance of primary osteoporosis in a quad-phasic non-contact injury pathway. Furthermore, it is emphasised that DOMS, NC-ACL injury, OA and osteoporosis could have the same initiating proprioceptive Piezo2 microinjury, at different locations, however, with different environmental risk factors and a different genetic predisposition, therefore producing different outcomes longitudinally. Taking a neural and interdisciplinary view could promote a better understanding of the pathomechanism of osteoporosis, which could result in even more precise therapeutic management in the future.

## Figures and Tables

**Table 1:** The quad-phasic non-contact injury model [5].

PIEZO2 MICROINJURY-INDUCED QUAD-PHASIC NON-CONTACT INJURY MODEL [5]				
**ENVIRONMENTAL FACTORS**		**PRIMARY INJURY PHASE**		**GENETICAL PREDISPOSITION**
	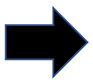	Repetitive superposition of compression forces	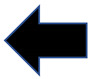	
		Fatigue-induced acute stress response		
	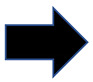	Acute stress-derived energy depletion of the mitochondria in the somatosensory terminal contributing to proprioception	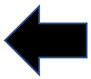	
		Mechano-energetic impairment of Piezo2		
		Painless compression Piezo2 channelopathy		
	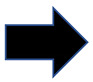	**SECONDARY INJURY PHASE**	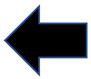	
		Harsher tissue damage due to impairment of Piezo2 with C-fibre contribution		
	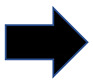	Painful compression axonopathy	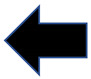	
		**TERTIARY INJURY PHASE**		
	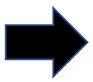	Re-injury and sensitisation could evolve into chronic condition and earlier aging due to repetitive overloading	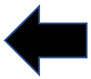	
		Chronic neuroinflammation or ganglionopathy		
	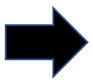	**QUADRIC INJURY PHASE**	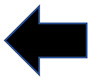	
		Aging or non-resolving neuroinflammation-induced Piezo2 microinjury or augmentation of former channelopathy and ganglionopathy		

## Data Availability

Not applicable.
